# Assessment of the Relationship between Antero-Posterior Dental Malocclusions, Body Posture Abnormalities and Selected Static Foot Parameters in Adults

**DOI:** 10.3390/jcm13133808

**Published:** 2024-06-28

**Authors:** Monika Nowak, Joanna Golec, Piotr Golec, Aneta Wieczorek

**Affiliations:** 1 Faculty of Medicine and Health Sciences, Andrzej Frycz Modrzewski Krakow University, 30-705 Kraków, Poland; 2 Institute of Clinical Rehabilitation, University School of Physical Education in Krakow, 31-571 Kraków, Poland; joannagolec@wp.pl; 3 Individual Medical Practice, 30-390 Kraków, Poland; piotrgolec88@gmail.com; 4 Department of Prosthodontics and Orthodontics, Faculty of Medicine, Jagiellonian University Medical College, 31-007 Kraków, Poland; aneta.wieczorek@uj.edu.pl

**Keywords:** body posture, head position, malocclusion, podal system, TMD

## Abstract

**Objectives**: This study aimed to find if a relationship exists between antero-posterior malocclusions and the level of musculoskeletal disorders in adults, including body posture and static foot analysis. **Methods**: In all, 420 participants were recruited through convenience sampling (Kraków University students and patients of a local dentist’s practice). Following general medical interviews, dental examinations and consideration of inclusion and exclusion criteria, 90 healthy volunteers (ages 19–35) were enrolled and assigned to three groups (*n* = 30) based on occlusion type (Angle’s molar Class I, II or III). The research procedure involved occlusion and temporomandibular disorder assessment conducted by a dental specialist. Comprehensive morphological measurements of body asymmetry were performed using the Videography 2D package and FreeSTEP software, which calculated the parameters determined from anterior, posterior and lateral projection photos. Foot loading distribution was analyzed using the FreeMED baropodometric platform. **Results**: Significant differences were demonstrated in the positioning of the head, cervical and lumbar spine in the sagittal plane among individuals with the analyzed occlusal classes (*p* < 0.05). Individuals with Angle’s Class II exhibited significantly greater forward head positions and greater depths of cervical and lumbar lordosis compared with individuals with Class III or Class I. Those with overbites had higher forefoot loading. The Class III individuals exhibited greater L-R displacement, indicating a larger angle of displacement of the centers of the right and left feet relative to the lower edge of the measurement platform, suggesting pelvic rotation. **Conclusions**: An inclination for concurrent occurrences of malocclusions and posture deviations in the sagittal plane was observed. An interdisciplinary approach involving dentistry and physiotherapy specialists which utilizes tools for comprehensive posture assessment is crucial for diagnosing and treating such conditions.

## 1. Introduction

The human body is a complex system of interacting elements where disorders in one organ or system can lead to the malfunctioning of others [1,2]. This coherence is crucial when considering body posture, defined as the natural, unforced and habitual upright position of the body in space. Posture development is individualized and heavily influenced by the structural and functional integrity of skeletal and myofascial components, whose tension is essential for maintaining proper alignment and movement of body segments or their displacement [1,2,3].

Numerous factors influencing human body posture have been documented, including genetic, environmental and lifestyle factors [4,5,6]. Recently, an increasing number of researchers have suggested that disorders of the stomatognathic system, such as malocclusions, may also affect body posture and stability [6,7,8,9,10]. It has even been demonstrated that artificially induced occlusal changes can have both immediate (observed within seconds) [11] and long-term effects on the musculoskeletal system [12]. However, research findings in this area are inconsistent [2,13]. Furthermore, there are significant controversies regarding whether the correlations observed in experimental studies have clinical significance.

The World Health Organization (WHO) identifies malocclusions as the third most prevalent oral health problem in adults, coming after dental caries and periodontal diseases [14]. Studies indicate that the prevalence of malocclusions in the general population is around 56% and increases with age [15]. The consequences of malocclusions are multifaceted, impacting the medical, economic and social dimensions. They can affect basic functions such as breathing and chewing and lead to psychosocial issues related to facial aesthetics, influencing one’s self-esteem and quality of life. Additionally, malocclusions can disrupt the harmony of oral and maxillofacial structures, potentially leading to broader musculoskeletal imbalances [16].

Researchers suggest that abnormalities in the craniofacial area may lead to alterations in the stimulation of proprioceptors, transmitting incorrect neural information to the reticular formation. This misinformation can then affect the muscles of the neck and shoulder girdle, propagating through neuromuscular-fascial chains to more distal areas. Over time, as compensatory mechanisms are exhausted, this can result in pain symptoms, misalignment of individual body segments and the perpetuation of postural and foot abnormalities [6,17,18,19].

Despite the growing body of research, most studies focused on analyzing single areas of the body [8,9,20,21,22]. There is still a lack of high-quality analyses considering a global assessment of posture. Research has also typically concentrated on temporomandibular disorders (TMDs) [23,24] or artificially induced occlusal changes [25,26]. These studies often face limitations such as a lack of control groups, incomplete sample descriptions, unequal group sizes and a limited number of tested parameters [27,28].

Given the increasing prevalence of malocclusions, it is imperative to evaluate their potential consequences, which can develop from a young age and progressively worsen, exerting long-term effects on the musculoskeletal system. Raising awareness among individuals with malocclusions, as well as dental and physiotherapy professionals, is crucial, highlighting the necessity for comprehensive diagnostic and therapeutic protocols.

Therefore, this study aimed to find if a relation exists between antero-posterior malocclusions and the level of musculoskeletal disorders in adults, including body posture and static foot analysis. Understanding these relationships can enhance clinical practices and improve patient outcomes by providing insights into the broader implications of dental malocclusions.

## 2. Materials and Methods

Three groups of subjects presenting different types of occlusions (Angle’s Class I, II and III) were subjected to the analysis of parameters characterizing global body posture and a static analysis of their feet. This research was carried out following the principles outlined in the Declaration of Helsinki. Participants were given thorough verbal and written explanations about the trial and provided consent to participate in this study. The study was approved by the ethics committee of the District Medical Chamber in Krakow University (reference number 35/KBL/OIL/2019; consent obtained on 19 February 2019).

This study was conducted from April 2020 to August 2021 at the Biomechanics Laboratory located within the Faculty of Medicine and Health Sciences at the Andrzej Frycz Modrzewski Krakow University. The study involved volunteers of Caucasian descent from the Lesser Poland Voivodeship aged 19–35.

A total of 420 participants were recruited through convenience sampling (including Kraków University students and patients of a local dental clinic) without prior selection. Information about the study was disseminated through informational posters placed in university campuses and the dental clinic, ensuring broad visibility and accessibility. Interested individuals were then provided with further details about the study. Upon expressing interest and consenting to participate, they underwent a general medical interview and dental examination to meet the inclusion and exclusion criteria for the study.

The inclusion criteria encompassed individuals aged between 18 and 35 years with Angle’s Class I, II, or III occlusions and possessing at least 28 permanent teeth. Exclusion criteria included the presence of braces, neurological or osteoarticular conditions or permanent consequences of traumatic musculoskeletal system injuries that could affect posture and balance.

Following screening and application of the above criteria, 90 individuals were included, consisting of 52 females (57.78%) and 38 males (42.2%).

The subjects were assigned to three groups based on the type of occlusion diagnosed during the dental examination using the molar Angle classification. The first group consisted of 30 individuals identified with Angle’s Class I (proper occlusions). The second group comprised 30 individuals diagnosed with Angle’s Class II, commonly referred to as distal occlusions. The third group included 30 individuals identified with Angle’s Class III, indicating anterior occlusions.

After detailing the participant recruitment process, this study proceeded to implement the research procedures, which involved dental examinations. The occlusions were evaluated through both intraoral and extraoral examinations using the molar Angle classification as previously mentioned. Angle’s classification assesses the correctness of an occlusion based on the position of the upper first molar relative to the lower dental arch [29]. Next, a functional examination of the masticatory system was conducted using the Research Diagnostic Criteria for Temporomandibular Disorders (RDC/TMD) protocol (Axes I and II). The examinations were performed by the same specialist in dental prosthetics, who was trained in and calibrated to the RDC/TMD procedure.

An assessment of the spatial positions of individual body segments was carried out using the Videography 2D package (Sensor Medica, Guidonia Montecelio, Italy), consisting of a camera, tripod and FreeSTEP 2.0 software (Sensor Medica, Guidonia Montecelio, Italy) used to collect, analyze and calculate the analyzed variables. A C920 Logitech HD Pro Webcam digital camera was used for digital photographic posture measurements. The camera captured images in Full HD quality (1080 pixels), enabling detailed analysis of each participant’s body posture. The camera with a tripod and laser spirit level was positioned at a distance of 170 cm from the examination site (participant’s position) and at the level of the umbilicus, following the manufacturer’s instructions for the FreeSTEP program.

**Figure 1:**
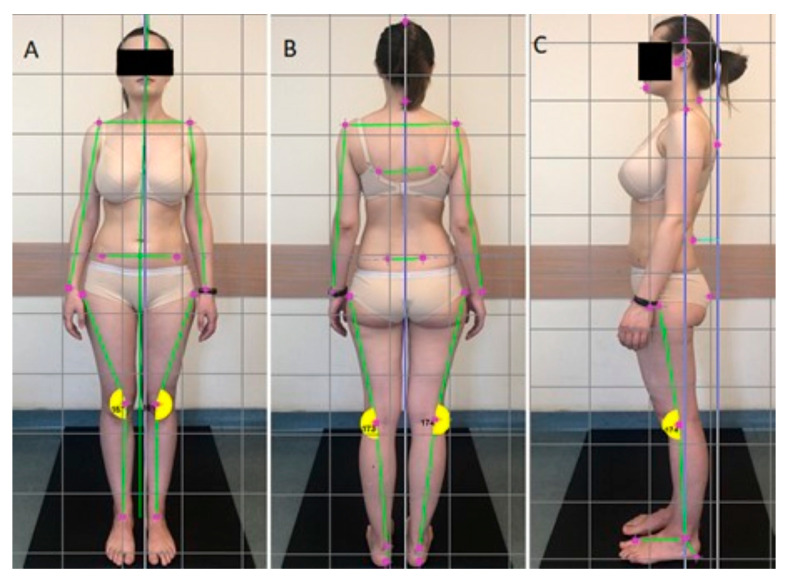
Parameters for the videographic examinations, determined by the FreeStep program in a photo taken from the anterior projection (**A**), posterior projection (**B**) and lateral projection (**C**, left side facing the camera).

The assessment of the spatial position of individual body segments was carried out by the same proficient researcher, ensuring consistency and reliability in the measurements. Prior to taking digital photographs, select bony landmarks were marked on the participants’ bodies (C7 spinous process, acromion processes of the scapulae, anterior superior iliac spines and posterior superior iliac spines), allowing for more precise identification of the same points on a computer after importing the images into the FreeSTEP software. Next, the participant stood at the photographic examination station. They were asked to assume a relaxed position, gaze straight ahead, place their feet hip-width apart and keep their upper limbs slightly bent at the shoulder joints at approximately 10–20°. Photographs of the participants’ body postures were taken under static conditions in anterior, posterior and lateral (left and right side) projections (Figure 1). To assess the postures and calculate potential asymmetries in each occlusion class, the same experienced researcher marked the required bony landmarks on the photographs using the FreeSTEP software as mentioned earlier. After importing the images into the FreeSTEP program and determining the bony landmarks on the computer, the program analyzed and calculated the following variables:

- In the sagittal plane, the depth of cervical lordosis, thoracic kyphosis and lumbar lordosis (in millimeters) were assessed (Figure 1). Additionally, a photographic measurement of the head protraction angle (in degrees) was performed, which is the angle between the line from the junction of cervical lordosis to thoracic kyphosis to the external auditory meatus and a horizontal line [30].
- In the frontal plane, the symmetry of shoulder alignment, pelvis alignment (relative to the horizontal line connecting the acromion processes of the scapulae and the anterior superior iliac spines) and deviation of the trunk and head (relative to the vertical line) were assessed (in degrees) (Figure 1).

**Figure 2:**
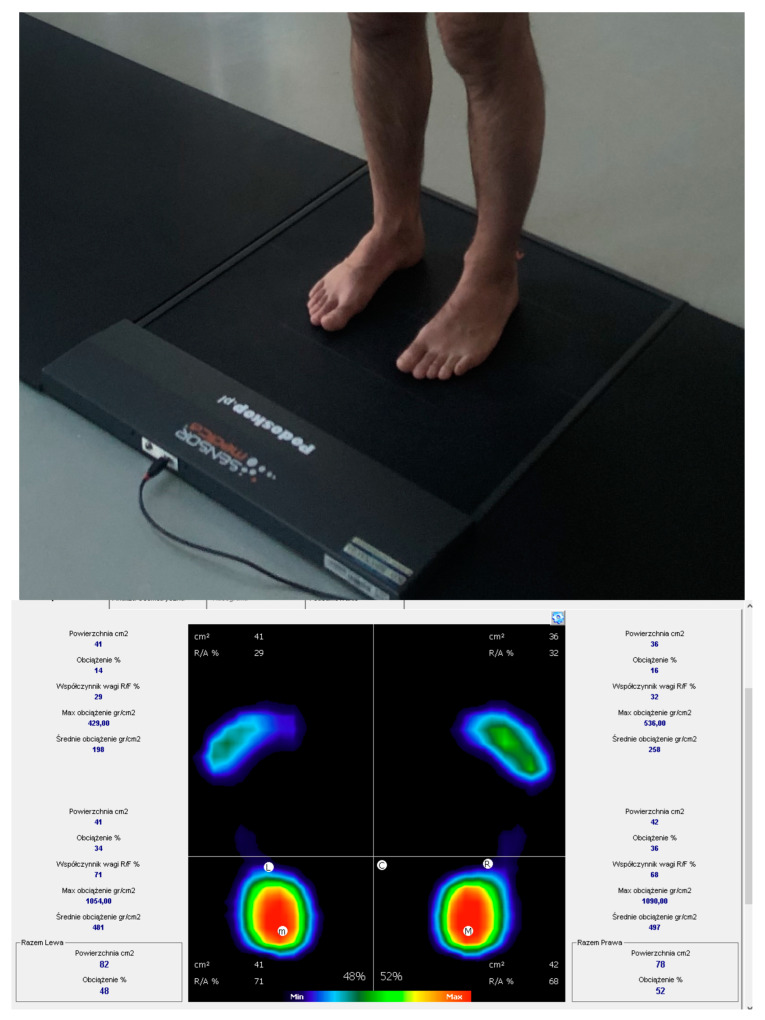
The measurement station equipped with a force platform along with a sample report from the conducted analyses.

The analysis of foot pressure distribution in static posture was conducted utilizing a baropodometric platform (FreeMED Base, Sensor Medica, Guidonia Montecelio, Italy), consisting of an active (40 × 40 cm) track with a sampling frequency of up to 400 Hz (Figure 2), along with the FreeSTEP 2.0 software. The measurement outcomes were presented in a software-generated report, which included details on the evaluated parameters.

The static analysis was conducted in a well-lit room with a temperature ranging between 21 and 22 °C. The subjects were instructed to stand barefoot on the measurement platform and assume a relaxed position, with their arms alongside their torsos and their feet hip-width apart. They were asked to fix their gaze on a point on the opposite wall. Three measurements of static foot analysis were conducted, followed by calculation of their average value. The static analysis was conducted in the habitual bite position, assessing parameters such as the percentage load on each foot, L-R displacement (in degrees) and the value of the plantar angle of the right and left foot (in degrees). The L-R displacement value represents the angle formed by the line connecting the resultant pressure points of the right and left foot and the lower edge of the measurement platform. According to the platform producers, this angle may indicate pelvic rotation. In turn, the plantar angle is formed by extending the line connecting the most protruding point of the forefoot and the rearfoot. In the literature, it corresponds to the gamma heel angle, where values ranging from 15° to 18° are considered normal [31].

Statistical calculations were conducted utilizing R software, version 4.1.1. The significance threshold was set at *p* < 0.05. The comparison of quantitative variables among the three groups was carried out using the Kruskal–Wallis test. In cases where statistically significant differences were observed, post hoc analysis with Dunn’s test was utilized to identify significantly distinct groups in terms of statistics. A comparison of the qualitative variables was conducted using a chi-squared test or Fisher’s exact test, where low expected counts appeared in the tables.

The homogeneity analysis of the sample was conducted using ANOVA and a chi-squared test. ANOVA was employed to assess the homogeneity of groups in terms of age, weight and height, while the chi-squared test was used to analyze the homogeneity of the groups concerning gender.

## 3. Results

The analysis of the ages and body structures of participants with various occlusal types (Angle’s Class I, II and III) indicated that both the ages and body proportions across these groups were similar. Basic data describing each group are shown in Table 1.

Based on the measurements provided by the RDC/TMD form, it was possible to diagnose and categorize the subjects’ symptoms into one of three TMD disorder groups: I (myofascial), II (disc displacement) or III (arthralgia, inflammation and degenerative joint disease of the temporomandibular joint (TMJ)). Correlation analysis revealed no significant relationship between the occlusion type and the occurrence of TMJ disorders (*p* > 0.05) at either the myofascial level or within the disc or TMJ.

The digital photographic posture analysis (Videography 2D) enabled the assessment of postures in the sagittal plane and the determination of the angle of head protraction (in degrees) and the depth of cervical lordosis, thoracic kyphosis and lumbar lordosis (in millimeters).

**Figure 3:**
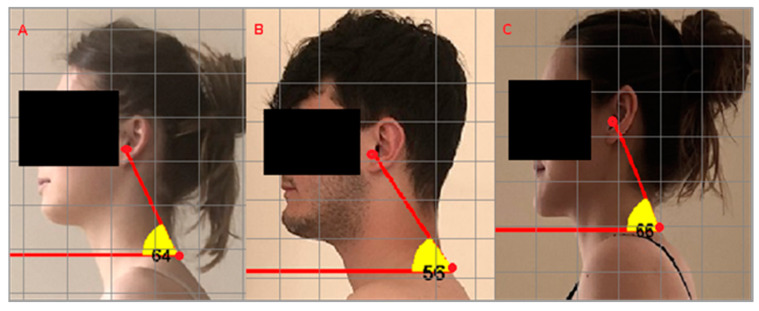
Marking points and exemplary measurement of the photographic head protraction angle in people with Angle’s Class I (**A**), II (**B**) and III occlusions (**C**).

The highest values of the head protraction angle were observed in individuals with Class III occlusions, and the lowest was found in those with Class II occlusions (Table 2). The smaller the head protraction angle, the greater the head’s position forward. Figure 3 illustrates examples of the photographic measurements and the size of the head protraction angle in individuals with Angle’s Class I, II and III. The analysis of the results for each group revealed that individuals with Class II occlusions exhibited the highest means both in terms of the depth of cervical lordosis (in millimeters) and lumbar lordosis, while those with Class III occlusions demonstrated the lowest means.

The statistical analysis of the results obtained during the posture photographic measurements revealed significant differences between the examined groups (*p* < 0.05). The angle of head protraction was significantly greater in individuals with Class I and III occlusions compared with those with Class II occlusions (*p* < 0.001) (Table 2). The depths of cervical and lumbar lordosis were significantly greater in the individuals with Class II occlusions compared with those with Class I and III occlusions (*p* < 0.05).

In the frontal plane, the following parameters were calculated: the positioning of the shoulder line and pelvis, as well as the positioning of the trunk and head in lateral flexion. An oblique position of the shoulders in relation to the ground occurred in the highest percentage (23.33%) in the people with Class II occlusions. The highest mean oblique position of the line connecting the scapular acromion in the frontal plane was observed in the subjects with Class III occlusions (Table 3). Asymmetric positioning of the horizontal line relative to the ground, connecting the anterior superior iliac spines, was most commonly observed in the individuals with Class II occlusions (56.66%). The highest mean values of inclination were noted in the group with proper occlusions and the lowest in the group with Class III occlusions (Table 3). The correct head positioning in the frontal plane was most frequently observed in people with proper occlusions (40%). In turn, the tendency for head deviation was most pronounced in the group with Class II occlusions (76.66%). The same pattern was observed in the analysis of the mean angle of head positioning in lateral flexion, where the highest values were also noted in the group with Class II occlusions and the lowest in individuals with Class I occlusions (Table 3).

There were no significant differences between the studied groups in all parameters analyzed in relation to the frontal plane (Table 3) as well as the direction of deviation and inclination of the assessed body areas (i.e., positioning of the shoulder (*p* = 0.127) and pelvis (*p* = 0.506) line, trunk (*p* = 0.621) and head (*p* = 0.714) deviation).

When using a platform to assess the ground reaction forces in terms of statics, the following parameters were analyzed: the percentage load distribution of the right and left foot and forefoot-rearfoot distribution (as a percentage), L-R displacement value (in degrees) and the values of the plantar angles of the right and left foot (in degrees).

As a result of the conducted research, the average values of the percentage load distribution on each foot among individuals with different occlusal classes were determined, as presented in Table 4. According to the FreeSTEP software, the results indicating proper load distribution fell within the range of 47–53%, with a maximum difference of 3% between the examined feet. Data analysis revealed correct load distribution only in 53.33% of individuals with proper occlusions, 50% with Class II occlusions and 43.33% with Class III occlusions.

When analyzing the distribution of loads in the forefoot and hindfoot among the subjects, it was shown that the highest average values of the percentage load on the forefoot were observed in people with Class II occlusions and the lowest in those with Class III occlusions. When examining the load on the hindfoot, the results obtained were inversely proportional (Table 4).

No significant relationships were found between the type of malocclusion and the overall percentage load on the right (*p* = 0.555) and left (*p* = 0.555) feet. Regarding the left forefoot, a significantly higher percentage load was observed in individuals with Class II occlusions compared with people with Class I and III occlusions. A significantly higher percentage load on the right forefoot was noted in individuals with Class II occlusions compared with those with Class I occlusions, while it was significantly higher in individuals with Class I occlusions compared with those with Class III occlusions. The load on the rearfoot for both lower limbs was significantly higher in individuals with Class III and I occlusions compared with those with Class II occlusions (*p* < 0.05) (Table 4).

The highest mean value of L-R displacement was observed in the group with Class III occlusions, while the lowest mean value was in those with Class I occlusions (Table 4). The norms for L-R displacement according to FreeSTEP software ranged from 0 to 2°. The correlation analysis between L-R displacement and malocclusion type did not reveal statistically significant differences between the examined groups (*p* > 0.05) (Table 4). Values above the norm were significantly more frequent in individuals with Class III occlusions compared with those with Class I occlusions (*p* = 0.049).

Then, the values of the plantar angles of the right and left feet were analyzed in individuals from each skeletal class. The highest average values of the plantar angle in the right and left foot occurred in people with Class II occlusions (Table 4).

The normative values ranged from 15 to 18 degrees. Values above 18 degrees, indicating the presence of flat feet or anterior displacement in foot loading, were predominantly observed in individuals with Class II occlusions, with 76.6% in the left foot and 83.3% in the right foot. In turn, values below 15 degrees, indicative of excessive supination or foot hypermobility, were predominantly found in the right feet of individuals with Class III occlusions (26.7%) and in the left feet of those with proper occlusions (23.3%). There were no significant differences between the analyzed groups in terms of the plantar angle of the right or left foot (*p* > 0.05).

## 4. Discussion

In accordance with current medical principles, which are aimed toward a holistic perspective of the human body, this interdisciplinary study aimed to assess the coexistence of malocclusions alongside disturbances in global body posture and abnormalities in the selected parameters of static foot analysis.

Certain tendencies toward the coexistence of anteroposterior malocclusions and abnormalities in sagittal plane body posture were demonstrated, as well as differences in foot load distribution. In summary, individuals with Angle’s Class II occlusions exhibited significantly greater forward head positions and greater depths of cervical and lumbar lordosis compared with individuals with Class III and I occlusions, characterized by a retracted head position. Biomechanical analysis of the feet in terms of statics revealed significantly higher percentages of forefoot loading in individuals with Class II occlusions compared with those with Class III and I occlusions. Values of L-R displacement above the norm were significantly more common in individuals with Class III occlusions than those with Class I occlusions.

The human body posture is commonly understood as the shape and position of its individual elements relative to each other in an unforced upright position [3]. In our own research, Videography 2D was used to assess the spatial positioning of individual body segments in people with malocclusions. This method relies on digital body photography techniques, enabling calculation of the basic quantitative features of human posture. This method is characterized by noninvasiveness, repeatability and measurement sensitivity [32]. In the reports of other authors, digital photography was used for evaluating the body posture of both children and adults with various dysfunctions and disorders [30,32,33]. In our own research, the use of modern software (FreeSTEP) allowed for precise calculation and analysis of the selected parameters, which is significant from the perspective of objectifying the obtained results [34].

The co-occurrence of disorders within the stomatognathic system with postural abnormalities has been the subject of previous studies, mostly being limited to analysis of the head and neck area [8,21,22,23,35]. There is a lack of analyses concerning comprehensive posture assessment encompassing various body segments and planes in individuals with specific malocclusions. In the presented studies, where body posture was evaluated in both the sagittal and frontal planes, it was observed that antero-posterior malocclusions result in deviations in body posture primarily in the sagittal plane. Furthermore, the presented results may suggest that if a malocclusion conditions or influences other body areas, then these interactions especially occur in regions directly related to and in close proximity to the stomatognathic system, namely the head and neck area. Clinicians have long demonstrated the existence of neuroanatomical connections between the oral cavity and the cervical spine, where afferents from the dental apparatus, masticatory muscles and temporomandibular joints converge at the trigeminal nucleus along with sensory information from the cervical spine [19,36]. According to Sforza [19], balanced tension between the structures of the head and neck is largely correlated with occlusion and jaw positioning. Occlusal disorders can lead to changes in dental proprioceptor stimulation, thus causing disorganization of the neck muscles and postural muscles as well as changes in head position [6,19].

In this study, the photographic head protraction angle was used to assess head positioning in the sagittal plane, employing the measurement method proposed by Bolzan et al. [30]. It was found that the photographic head protraction angle was significantly smaller in individuals with Angle’s Class II occlusions compared with those with Class I and III occlusions, suggesting that individuals with Class II occlusions exhibited greater forward head postures. The depths of cervical lordosis and lumbar lordosis were also significantly greater in individuals with Class II occlusions. Clinicians suggest that excessive forward head positions in individuals with Class II occlusions, characterized by a retrognathic mandibular position relative to the maxilla, may be dictated by the need to open the upper airway in this manner to enhance its functional capacity [37]. Others have suggested that anterior positioning of the human head reduces the field of vision, and attempts to improve the field of vision increase lordosis in the cervical spine [38]. The results of our own research are supported by the cited reports, as individuals with Class II occlusions were observed to have a significantly higher incidence of both of these features (anterior head positioning and increased lordosis in the cervical spine). Bricot [39] also reported that individuals with Class II occlusions are characterized by anterior positioning of the head and shoulders, unlike prognathic individuals. The author explained this as an attempt to balance the body in response to afferent information emitted by the position of the temporomandibular joint [39]. In reports by Sandoval et al. [8] and D’Attilio et al. [21], it was also demonstrated that individuals with Class II occlusions exhibited significantly greater anterior head protrusion than those with Class I or III occlusions, and in individuals with Class III occlusions, similar to the findings of this study, flattening of the cervical lordosis was observed significantly more frequently. Deda et al. [22], like in the present study, compared head positions in different malocclusions using photogrammetry and a significantly smaller sample size than that in the present study. During clinical examination, they observed a pattern in the head positions of individuals with Class II occlusions where, as reported, 100% had their heads protruded forward, while in the Class I group, 73.3% had a neutral head position. Lippold [40] conducted an analysis of individual spinal segments in relation to the morphology of the craniofacial complex in individuals with Class II and III occlusions, demonstrating differences in pelvic tilt and the size of the lumbar lordosis angle between the examined groups. Similarly, in our own studies, significant differences in the depth of lumbar lordosis were also demonstrated, depending on the Angle’s classification.

In our own research, an attempt was made to assess the relationship between the stomatognathic system and the biomechanical characteristics of the feet, where the selected parameters of static examination, including load distribution, L-R displacement and the plantar angle of the feet, were evaluated in individuals with various types of malocclusions.

Valentino et al. [20], in a study based on electromyography, observed a correlation between the occlusal plane and the muscles supporting the foot arches. Marchena-Rodríguez et al. [41] demonstrated that Clarke’s angle, which determines the state of longitudinal arch of the foot, tends to decrease with an increase in malocclusion classification from Class I to III. In our own research, the shape of the transverse arch of the feet was assessed by determining the value of the plantar angle, but no significant differences were found between the examined types of malocclusion. On the other hand, Cuccia [42] conducted studies using a podobarographic platform and compared footprints between individuals with TMD and healthy controls. They demonstrated differences in the foot arches between both groups and also observed changes in the distribution of load on the forefoot and rearfoot after artificially inducing occlusal imbalance. However, the authors did not find a relationship between the percentage load on the lower limbs and different occlusal conditions. The results of our own research in this regard are consistent with the findings of Cuccia [42], where no significant differences were found between various types of malocclusions and the percentage load on the lower limbs in statics. However, significant differences were observed in the load on the forefoot and rearfoot depending on the occlusion, with individuals with Class II occlusions showing significantly higher percentages of load on the forefoot compared with those with proper occlusion and Class III occlusions. Similarly, a significantly higher percentage load on the rearfoot was noted in individuals with Class III and I occlusions. In our own studies, tendencies for the forward head posture were observed in the same group in which a significantly higher percentage load on the forefoot was recorded in static analysis, namely individuals with Angle’s Class II occlusions. The same observation applied to individuals with Class III occlusions, where a significantly more frequent occurrence of head retraction and a higher percentage load on the rearfoot were observed compared with individuals with Class II occlusions. This may suggest that the changes observed in the feet in terms of load distribution could be due to the positions of the head and neck, which according to some authors influence the position of the body’s center of gravity [4]. The L-R displacement, which represents the angle formed by the connection of the resultant points of pressure forces for the left and right foot and the lower edge of the platform, was significantly more frequently above the norm in individuals with Class III occlusions, which according to the FreeSTEP software manufacturer may indicate pelvic rotation in the subjects [43]. Lippold et al. [44] also demonstrated correlations between the morphology of the craniofacial region in adults, specifically the presence of Class II and III occlusions and pelvic rotation.

This study, in addition to its primary objectives, aimed to emphasize the significance of the stomatognathic system in the context of the proper functioning of other areas of the body, which are part of the human musculoskeletal system. Its goal was also to present novel approaches for diagnosing and treating various dental disorders and postural disturbances. It would be beneficial to develop an interdisciplinary treatment protocol which could facilitate early diagnosis and therapy. Considering the results of our own research and reports from other authors, a holistic model of human assessment and evaluation of the craniofacial area in the overall functional examination of a patient is recommended. This integrated perspective may lead to better outcomes for therapeutic interventions.

Despite providing significant data regarding the relationship between Angle’s classification and body posture (taking into account a comprehensive assessment of individual body segments and planes) as well as select parameters of static foot examination which, when combined, fill a gap in the available scientific research, these studies have certain limitations that need to be considered. Firstly, the sample size (*n* = 90) was relatively small, limiting the ability to generalize the results to a larger population. Secondly, our study was based on assessing malocclusions in a molar relationship. Typically, relationships between malocclusions and associated health outcomes are assessed using skeletal classification, which better reflects jaw misalignments than dental assessments. However, as our study represents a preliminary analysis, we opted to utilize dental classification due to its wide applicability, availability and technical feasibility. It serves as a preliminary exploration which may pave the way for more advanced research incorporating skeletal classification assessed through cephalometry. Additionally, the results of the presented studies may suggest the presence of certain tendencies in individuals with malocclusions, postural disorders and changes in foot loading in statics. Nevertheless, it is important to note that these studies did not explain and could not determine the causality of the observed associations. A more detailed analysis is needed with a larger sample size using electromyography, spinal radiographs or advanced motion analysis systems to better determine the relationships between these variables.

## 5. Conclusions

In conclusion, the obtained results indicate tendencies toward the co-occurrence of antero-posterior malocclusions and deviations in sagittal plane body posture, encompassing the area of the head and spine, as well as differences in foot loading distribution, depending on the Angle’s classification. These preliminary findings highlight the importance of considering both body posture and malocclusion in diagnosing and treating orthopedic and dental patients. A multidisciplinary approach involving dentists and physiotherapy specialists is necessary, with the development of shared diagnostic protocols and the utilization of tools for comprehensive body posture assessment. This study opens the way to investigating whether orthodontic treatment affects body posture.

## Figures and Tables

**Table 1:** Basic anthropometric characteristics of the analyzed groups, including individuals with Angle’s Class I, II, and III.

Whole Group (*n* = 90)	Angle’s Class I (*n* = 30) Average (SD)	Angle’s Class II (*n* = 30) Average (SD)	Angle’s Class III (*n* = 30) Average (SD)	*p*
Gender	17♀/13♂	16♀/14♂	19♀/11♂	0.225
Age (years)	22.63 (2.65)	23.87 (3.90)	22.77 (2.24)	0.263
Height (cm)	170.93 (10.10)	172.8 (10.0)	171.73 (11.20)	0.590
Body mass (kg)	68.23 (12.9)	66.93 (11.93)	67.27 (12.20)	0.727

*p*: chi-squared test (gender); ANOVA (age, height and body mass).

**Table 2:** Selected parameters for videographic examination in the sagittal plane in people with particular types of occlusions.

Parameters		Angle’s Class			*p*
		I	II	III	
Depth of cervical lordosis (mm)	Average (SD)	67.83 (18.41)	79.03 (14.28)	66.07 (17.38)	*p* = 0.006 *
	Median	69	77.5	65	
	Quartiles	60–78.5	69.25–86	55–78.5	II > I, III
Depth of thoracic kyphosis (mm)	Average (SD)	2.9 (9.81)	3.57 (7.66)	3.4 (8.14)	*p* = 0.491
	Median	0	0	0	
	Quartiles	0–0	0–0	0–0	
Depth of lumbar lordosis (mm)	Average (SD)	54.67 (12.21)	64.23 (16.07)	51.2 (10.69)	*p* = 0.001 *
	Median	54.5	62	52.5	
	Quartiles	48.25–59	54.5–68.75	47–55.75	lI > I, III
Angle of head protraction (degree)	Average (SD)	62.13 (3.91)	58.8 (3.25)	62.37 (3.17)	*p* < 0.001 **
	Median	62	59	62.5	
	Quartiles	60–64	56–61.75	60–66	III, I > II

* Significant at *p* < 0.05. ** Significant at *p* < 0.001. *p*: Kruskal–Wallis test + post hoc analysis (Dunn’s test). SD = standard deviation. I, II and III are in relation to Angle’s classes.

**Table 3:** Selected parameters of videographic examination for the frontal plane in people with Angle’s Class I, II and III occlusions.

Parameters		Angle’s Class			*p*
		I	II	III	
Oblique shoulder line positioning (degrees)	Average (SD)	1.37 (0.89)	1.3 (0.99)	1.73 (0.98)	*p* = 0.151
	Median	1	1	2	
	Quartiles	1–2	1–2	1–2	
Oblique pelvic position (degrees)	Average (SD)	0.73 (0.78)	0.67 (0.66)	0.6 (0.89)	*p* = 0.581
	Median	1	1	0	
	Quartiles	0–1	0–1	0–1	
Lateral inclination of the torso (degrees)	Average (SD)	89.27 (0.94)	89.43 (1.07)	89.77 (0.77)	*p* = 0.161
	Median	89	90	90	
	Quartiles	89–90	89–90	89–90	
Lateral head tilt (degrees)	Average (SD)	1.03 (1.25)	1.67 (1.37)	1.5 (1.43)	*p* = 0.123
	Median	1	2	2	
	Quartiles	0–1	1–2	0–2	
Asymmetric positioning of the lower blade angles (degrees)	Average (SD)	1.63 (1.16)	1.77 (1.52)	1.73 (1.14)	*p* = 0.954
	Median	1.5	1	1.5	
	Quartiles	1–2	1–3	1–2	

*p*: Kruskal–Wallis test + post hoc analysis (Dunn’s test). SD = standard deviation.

**Table 4:** Results of selected parameters of static foot examination in subjects with Angle’s Class I, II, and III occlusions.

Parameters		Angle’s Class			*p*
		I	II	III	
Percentage load LF (%)	Average	50.90	49.23	50.27	*p* = 0.555
	Mediana	51	49.5	52	
	Quartiles	47.25–53.75	46.5–53	46.25–55	
Percentage load RF (%]	Average	49.10	50.77	49.73	*p* = 0.555
	Median	49	50.5	48	
	Quartiles	46.25–52.75	47–53.5	45–53.75	
Forefoot percentage weight-bearing LF (%)	Average	36.73	45.53	31.43	*p* = 0.002 *
	Median	36	42.5	30	
	Quartiles	27.25–43.75	35.5–52	21–40.25	II > I, III
Forefoot percentage weight-bearing RF (%]	Average	37.1	46.7	29.57	*p* < 0.001 **
	Median	38	46	28	
	Quartiles	29.25–44.5	35.25–57.75	21.25–36	II > I > III
Hindfoot percentage weight-bearing LF (%)	Average	63.27	54.47	67.07	*p* = 0.004 *
	Median	64	57.5	70	
	Quartiles	56.25–72.75	48–64.5	57.5–79	III, I > II
Hindfoot percentage weight-bearing RF (%]	Average	62.90	53.30	68.60	*p* < 0.001 **
	Median	62	54	71	
	Quartiles	55.5–70.75	42.25–64.75	61.75–74.75	III, I > II
L-R displacement (degrees)	Average	2.83	3.53	3.67	*p* = 0.416
	Median	3	3	3	
	Quartiles	0–4	0–6	3–4	
Plantar angle LF (degrees)	Average	16.17	19.30	16.77	*p* = 0.058
	Median	15.5	19	16.5	
	Quartiles	13.25–19	15–21	14.25–19.5	
Plantar angle RF (degrees)	Average	17.73	18.53	16	*p* = 0.079
	Median	17	19	16.5	
	Quartiles	14–20.7	16–21	12.25–18	

* Significant at *p* < 0.05. ** Significant at *p* < 0.001. *p*: Kruskal–Wallis test + post hoc analysis (Dunn’s test); LF = left foot; RF = right foot; I, II and III are in relation to Angle’s classes.

## Data Availability

Data are contained within the article.
